# Emerging antibacterial nanozymes for wound healing

**DOI:** 10.1002/SMMD.20220025

**Published:** 2023-02-19

**Authors:** Jingyang Shan, Junyi Che, Chuanhui Song, Yuanjin Zhao

**Affiliations:** ^1^ Department of Rheumatology and Immunology Nanjing Drum Tower Hospital State Key Laboratory of Bioelectronics School of Biological Science and Medical Engineering Southeast University Nanjing China; ^2^ Department of Neurology Shenzhen Institute of Translational Medicine The First Affiliated Hospital of Shenzhen University Shenzhen Second People's Hospital Guangdong Key Laboratory for Biomedical Measurements and Ultrasound Imaging School of Biomedical Engineering School of Medicine Shenzhen University Shenzhen China

**Keywords:** bacteria, nanozymes, reactive oxygen species, skin regeneration, wound

## Abstract

Wound infections continuously impose a huge economic and social burden on public healthcare. Despite the effective treatment of bacteria‐infected wounds after using traditional antibiotics, the misuse of antibiotics usually causes the spread of bacterial resistance and decreases therapeutic outcomes. Therefore, the development of efficient antibacterial agents is urgently needed. Nanozymes, as a new generation of artificial enzymes, combine the intrinsic abilities of nanomaterials and natural enzymes. Recently, nanozymes has been widely developed to kill bacteria and treat wound infections by catalyzing the generation of various reactive oxygen species. Thus, this new concept of “antibacterial nanozymes” will promote the further advances of connecting nanozymes and bacterial elimination. To highlight these achievements, we summarize different types of antibacterial nanozymes for wound healing. It is believed that such a promising therapeutic strategy of developing antibacterial nanozymes will make a great contribution in the field of skin regeneration. We expect that antibacterial nanozymes will play the significant roles in both basic research and clinical applications.

## Graphical abstract

Wound infections continuously impose a huge economic and social burden on public healthcare. Despite the effective treatment of bacteria‐infected wounds after using traditional antibiotics, the misuse of antibiotics usually causes the spread of bacterial resistance and decreases therapeutic outcomes. Therefore, the development of efficient antibacterial agents is urgently needed. Nanozymes, as a new generation of artificial enzymes, combine the intrinsic abilities of nanomaterials and natural enzymes. Recently, nanozymes has been widely developed to kill bacteria and treat wound infections by catalyzing the generation of various reactive oxygen species (ROS). Thus, this new concept of “antibacterial nanozymes” will promote further advances of connecting nanozymes and bacterial elimination. To highlight these achievements, we summarize different types of antibacterial nanozymes for wound healing. It is believed that such a promising therapeutic strategy of developing antibacterial nanozymes will make a great contribution in the field of skin regeneration. We expect that antibacterial nanozymes will play the significant roles in both basic research and clinical applications.

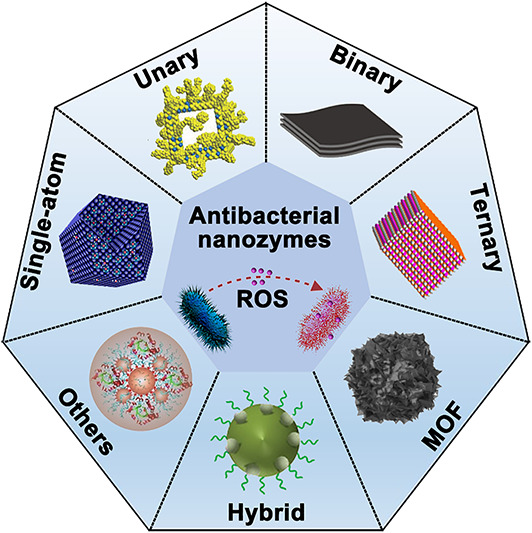

1

**Key points**

- The characteristics of antibacterial nanozymes are dissertated.
- The classes and properties of antibacterial nanozymes are discussed.
- The recent advances of antibacterial nanozymes in wound healing are reviewed.

## 2 INTRODUCTION

Bacteria‐induced wound infections have been continuously posing an immerse burden on public health around the world for decades.^1^, ^2^, ^3^ Among various treatment approaches, antibiotics are the most common therapeutics against bacterial infections. However, the main issue restricting the usage of antibiotics is bacterial resistance, which causes 700,000 people deaths annually due to the abuse of antibiotics, and the number will reach to 10 million in 2050.^4^, ^5^, ^6^, ^7^, ^8^ Furthermore, modern studies of novel antibiotics have stagnated because of high cost and long development time.^9^ Therefore, the exploration of new antibacterial alternatives is of great importance. In our living body, natural enzymes can catalyze various substrates to produce a series of reactive oxygen species (ROS) for fighting bacterial invasion.^10^, ^11^, ^12^ Nevertheless, their intrinsic shortcomings including high cost, poor stability, and limited possibility to scale up for manufacturing greatly hamper their further usage as antibacterial agents.^5^, ^12^, ^13^, ^14^ Thus, the efficient antibacterial agents aiming for clinical translations need to be explored.

Since iron oxide nanoparticles as peroxidase (POD) mimic were discovered in 2007,^15^ “nanozymes” were first defined as “nanomaterials with enzyme‐mimicking characteristics” in 2013 and then many nanozymes have attracted broad attentions because of their ease of production, low cost, and possibilities of multifunctionality.^5^, ^16^, ^17^, ^18^, ^19^, ^20^ With the recent advances of nanotechnology, nanozymes have revolutionized previous fundamental understandings in the field of biology, chemistry, and medicine.^18^, ^19^, ^20^, ^21^, ^22^, ^23^ At present, several nanozymes, including carbon‐based nanomaterials,^13^, ^18^, ^24^, ^25^ noble metal nanomaterials,^26^ transition metal‐based compounds nanomaterials,^27^ metal organic framework (MOF)‐related nanomaterials,^5^, ^28^, ^29^ and single‐atom nanozymes,^30^, ^31^ have been extensively studied in biomedicine for diagnostics by catalytic generation of ROS, including O_2_^•−^, H_2_O_2_, ^1^O_2_, and hydroxyl radical (•OH).^5^ For instance, noble metal nanomaterials can mimic oxidase (OXD) to catalyze some substrates (e.g., ascorbic acid) and oxygen for generating H_2_O_2_ ^26^; transition metal‐based compound nanomaterials as POD mimic are able to catalyze H_2_O_2_ to produce •OH.^22^, ^27^ These ROS can cause bacterial death by mainly destroying the cytoplasmic membrane, bacterial integrity, proteins, DNA, and so on.^5^, ^17^ Many excellent reviews have been published to summarize the significance and impact of nanozymes in the aspect of therapeutic applications, while the specific topic of antibacterial nanozymes for the treatment of wound healing has rarely been discussed.

**FIGURE 1:**
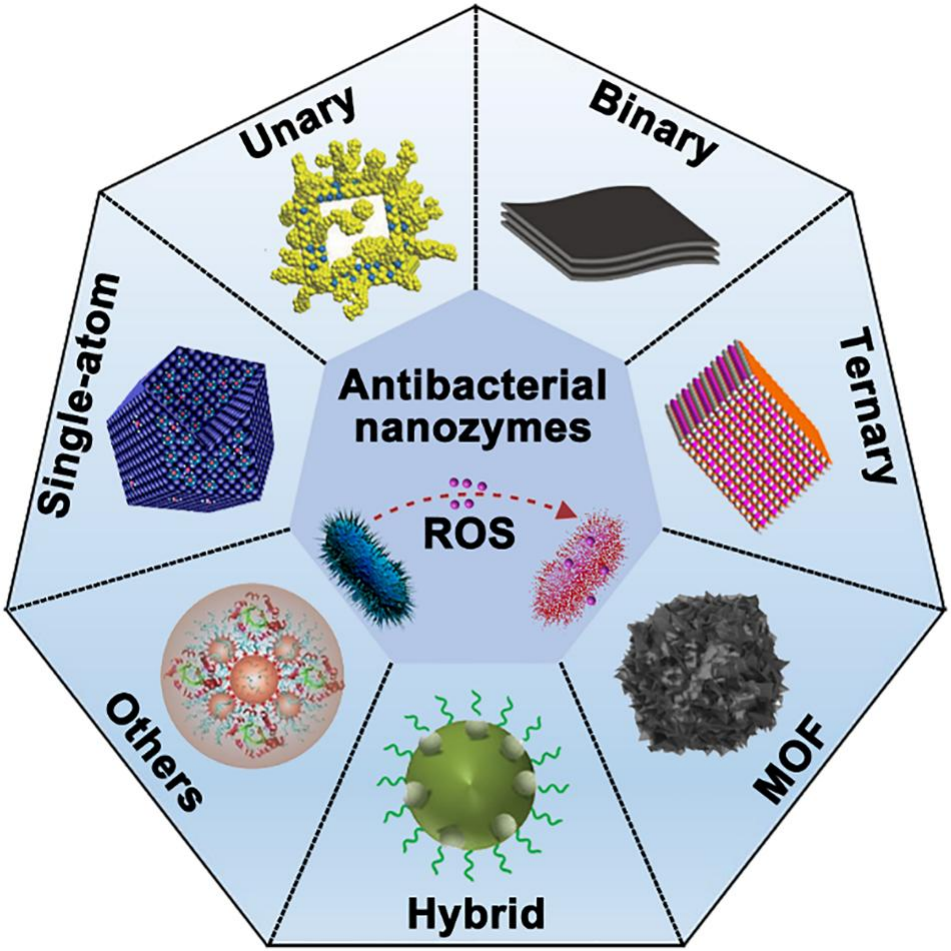
Illustration of antibacterial nanozymes in wound healing applications. MOF, metal organic framework; ROS, reactive oxygen species. *Source*: Images reprinted with permission from Refs.^32^, ^33^, ^34^, ^35^, ^36^, ^37^, ^38^ Copyright 2021, Elsevier B.V. on behalf of KeAi Communications Co. Ltd. Copyright 2018, WILEY‐VCH Verlag GmbH & Co. KGaA, Weinheim. Copyright 2018, American Chemical Society. Copyright 2019, American Chemical Society. Copyright 2016, WILEY‐VCH Verlag GmbH & Co. KGaA, Weinheim. Copyright 2022, Wiley‐VCH GmbH. Copyright 2021, Wiley‐VCH GmbH.

In the review, we summarize current emerging antibacterial nanozymes for treating wound infections. The rapid growth of research in antibacterial applications of nanozymes inspires us to present the concept of “antibacterial nanozymes,” which is herein defined as “nanomaterial‐based artificial enzymes with antibacterial activity.” Considering different classes of these antibacterial nanozymes for wound infection management, we then particularly introduce recent studies of antibacterial nanozymes based on the element composition of nanozymes (Figure 1). The classification of nanozymes based on the element composition will be able to help researchers rapidly read antibacterial nanozymes, know well the current status, and design novel antibacterial nanozymes.

## 3 SINGLE‐ATOM NANOZYMES FOR WOUND HEALING

Single‐atom nanozymes (SAzymes) combine the single‐atom technology with enzyme‐mimic catalytic sites, which provide great understandings for the catalytic mechanisms between nanomaterials and natural enzymes.^39^, ^40^, ^41^, ^42^ Reducing the size to single‐atom level of metal nanoparticles will enhance the utilization efficiency and catalytic abilities of metal nanomaterials.^43^, ^44^, ^45^, ^46^, ^47^, ^48^ Zhang et al. reported a dispersing isolated Pt atoms on FeO_x_ as catalysis to oxidize CO and presented the concept of “single‐atom catalysis.”^49^ Swiftly, many SAzymes, including single‐atom Pd nanozyme, single‐atom Fe‐containing nanozyme, and Fe‐N‐C nanozyme, were studied in environmental protection, medical treatment, and sensing.^50^, ^51^, ^52^ In this section, we summarized some antibacterial SAzymes for treating wound infection.

**FIGURE 2:**
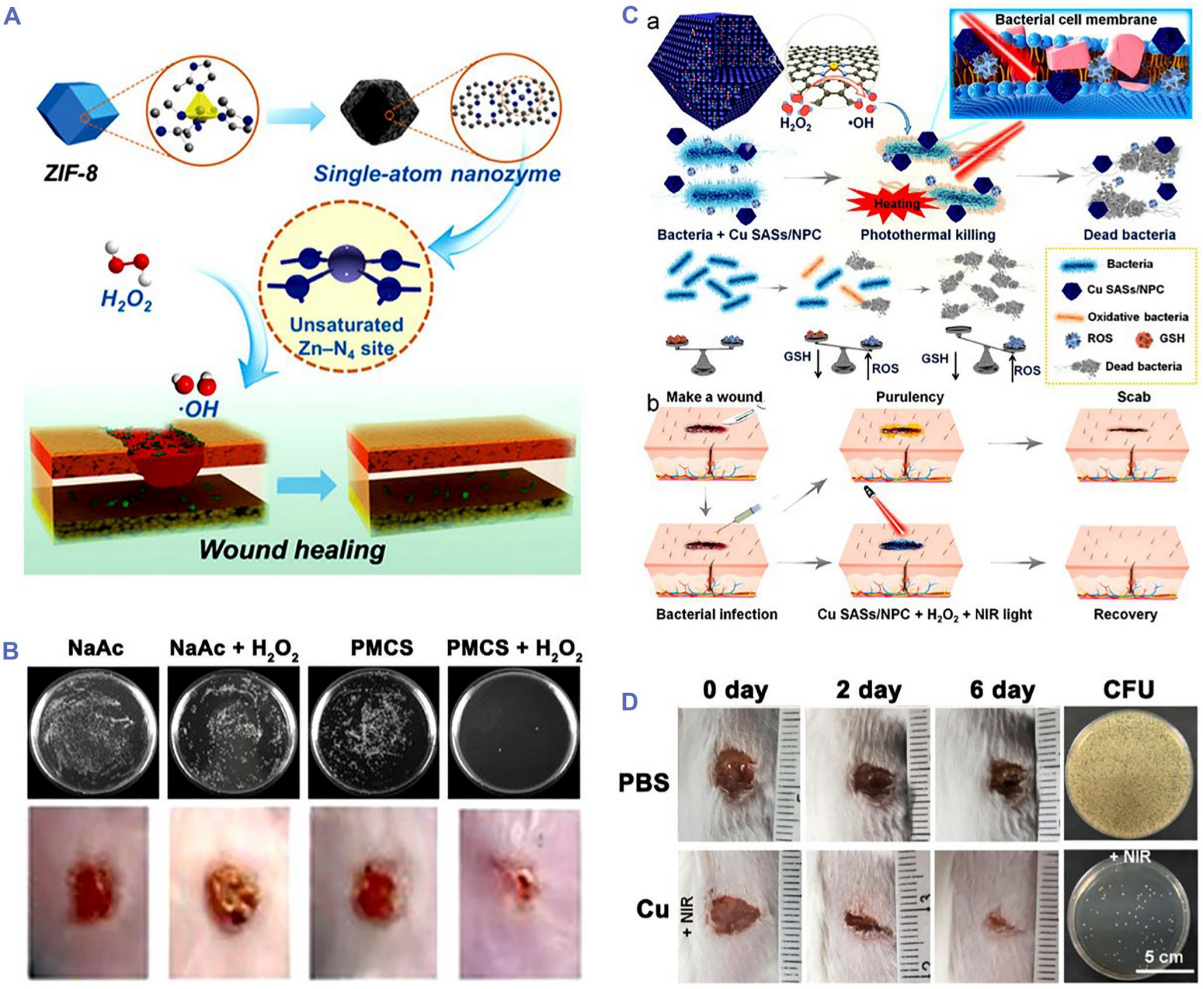
(A) Scheme of Zn‐based SAzyme with POD‐mimicking property for wound treatment. (B) Photographs of *Pseudomonas aeruginosa* colonies and infected wounds treated by different groups. *Source*: Images reprinted with permission from Ref.^53^ Copyright 2019, WILEY‐VCH Verlag GmbH & Co. KGaA, Weinheim. (C) Cu‐based SAzyme with enzyme‐like and photothermal properties for eradicating bacteria (a) and treating MRSA‐infected wounds (b). (D) Photographs of infected wounds and bacterial colonies from the tissues treated by different groups. *Source*: Images reprinted with permission from Ref.^32^ Copyright 2021, Elsevier B.V. on behalf of KeAi Communications Co. Ltd. NIR, near‐infrared; ROS, reactive oxygen species

In 2019, Xu et al. proposed a peroxidase (POD)‐like zinc‐based SAzyme with antibacterial ability for the promotion of wound healing (Figure 2A).^53^ The density functional theory (DFT) calculation demonstrated that the coordinatively unsaturated Zn–N_4_ sites could serve as the POD‐like activity of SAzyme. The SAzyme showed the higher catalytic activity than other carbon materials and Fe_3_O_4_ nanozyme at the same condition. Moreover, the SAzyme not only catalyzed the decomposition of H_2_O_2_ to generate •OH for inhibiting bacteria in vitro, but also promoted wound healing in vivo. The antibacterial test showed that the inhibition ratio of the SAzyme was 99.87% against *Pseudomonas aeruginosa* with addition of H_2_O_2_ using the plate counting method. The SAzyme with 100 μM H_2_O_2_ displayed complete wound repair in mice after 6 days of treatment, while the control group needed much longer healing period (Figure 2B). In addition, Wang et al. successfully prepared a copper‐based SAzyme for catalytic/photothermal antimicrobial treatment (Figure 2C).^32^ It was demonstrated that copper doping effectively improved the POD‐like activity of SAzyme than the non‐copper‐doped SAzyme. The combination of POD‐mimic ability and near‐infrared (NIR) photothermal property of the copper‐based SAzyme could catalyze H_2_O_2_ to generate •OH and induced hyperthermia resulting in bacterial death. Interestingly, the copper‐based SAzyme with glutathione POD‐like ability could consume glutathione in bacteria, which largely enhanced their antibacterial performance. The antibacterial efficiency of the copper‐based SAzyme was ∼100% against *Escherichia coli* (*E. coli*) and methicillin‐resistant *Staphylococcus aureus* (MRSA) owing to the photothermal/catalytic synergistic function. In vivo experiments verified that the copper‐based SAzyme efficiently healed MRSA‐infected wounds under NIR light irradiation (Figure 2D).

In recent studies, other metal‐based SAzymes were explored for the biomedical field, and some of them were studied for wound disinfection applications.^47^, ^48^, ^52^, ^54^, ^55^, ^56^, ^57^ Xu et al. fabricated single‐atom iron nanozymes for bacteria‐infected wound administration.^54^ The iron SAzyme with the POD‐mimic activity could generate •OH by adding H_2_O_2_ and showed NIR photothermal performance for producing heat and glutathione oxidization. Encouraged by the synergistic effect of these properties, iron SAzyme could efficiently kill bacteria in the presence of H_2_O_2_ in vitro and successfully treated *E. coli*‐infected wounds. This work provided a good example of a biofilm microenvironment‐activated iron SAzyme for antibacterial treatment and wound healing. Subsequently, Xu et al. reported a manganese (Mn) SAzyme for catalytic/photothermal treatment of bacteria‐infected wounds.^57^ The Mn SAzyme with the POD‐mimic activity could catalyze the generation of •OH in adding H_2_O_2_, and its catalytic activity was significantly improved by the NIR photothermal effect. Antibacterial assays showed that the synergetic catalytic and photothermal treatment achieved good antibacterial performances against *E. coli* and *Staphylococcus aureus* (*S. aureus*). In vivo bacteria‐infected wounds model showed that Mn SAzyme could inhibit inflammatory responses and promote wound healing, which would be a potential antibacterial nanozyme for skin regeneration. Apart from this, Huang et al. prepared a multifunctional and intelligent Pd SAzyme by integrating photosensitive polyazobenzene and sodium nitroprusside.^56^ The released NO upon ultraviolet (UV) light and POD‐like activity of Pd SAzyme exhibited the synergistic antibacterial effect with the addition of H_2_O_2_. The bacteria were completely eliminated at the concentration of 128 μg/ml under UV light irradiation. Antibacterial mechanism studies proved that the bacterial killing by the Pd SAzyme was attributed to the synergistic effect of produced ROS and NO. Additionally, the Pd SAzyme showed 78.3% wound‐healing ratio against *S. aureus*‐infected wounds under UV light on day 8. This work provided a synergistic modality to realize the nanozyme activity and NO storage in wound infection management. Despite some advances that SAzymes have made in the wound care, the current developed antibacterial SAzymes mainly focused on POD mimic, while SAzymes with other enzyme‐like activity, such as oxidase, have been rarely reported.

## 4 METAL AND METAL‐BASED COMPOUNDS NANOZYME FOR WOUND HEALING

### 4.1 Unary nanozymes

Unary nanozymes are metal‐based nanomaterials with enzyme‐mimic catalytic activity (such as Pt, Au, and Pd), exhibiting a strong catalytic activity due to their large surface metal atom ratios, and have been applied in biomedicine.^26^, ^58^, ^59^, ^60^, ^61^, ^62^, ^63^ Among them, Au nanoparticles (NPs) possess good antibacterial performance due to their special physicochemical properties.^17^, ^64^, ^65^ Yang et al. prepared small molecule‐coated Au nanoparticles (Au NPs) to kill various bacteria for treating bacterial wound infection.^66^ In 2015, Tao et al. reported a type of mesoporous silica‐supported Au NPs with dual enzyme‐like properties for antibacterial treatment.^64^ These silica‐supported Au NPs with POD‐like activity could covert H_2_O_2_ into •OH, while their oxidase (OXD)‐like ability was able to produce ROS. Although Au NPs have been demonstrated excellent antibacterial activity, Au nanomaterials as enzyme mimic applied for wound healing were rarely studied. In 2018, Fang et al. reported Pd nanocrystals with OXD‐ and POD‐mimicking properties against bacteria.^67^ Pd octahedrons with (111) facet showed lower catalytic activities than Pd cubes with (100) facet. DFT calculations showed that the homolytic dissociation of the adsorbed H_2_O_2_ entity on the Pd (100) facet was more favorable than that on the Pd (111) facet, which was consistent to the higher enzyme‐like catalytic activities of (100) faceted Pd cubes. Thus, Pd cubes achieved higher antibacterial activity by generation of more ROS than Pd octahedrons. Their studies firstly discovered the facet‐dependent enzyme‐like and antibacterial activities of noble metal nanozymes.

**FIGURE 3:**
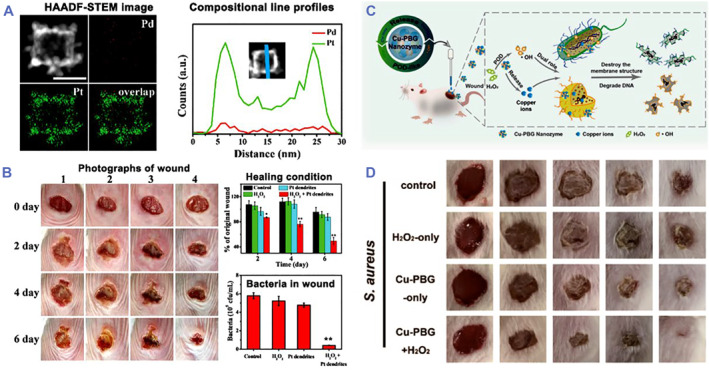
(A) Morphology and composition characterization of Pt hollow nanodendrites without Pd atoms. (B) Photographs of wound, wound healing condition, and bacterial colony forming unit in wound tissue treated by different groups. *Source*: Images reprinted with permission from Ref.^33^ Copyright 2018, WILEY‐VCH Verlag GmbH & Co. KGaA, Weinheim. (C) Copper‐doped nanozymes with POD‐like activity for treating wound infection. (D) Photographs of *S. aureus*‐infected wounds treated by copper‐doped nanozymes. *Source*: Images reprinted with permission from Ref.^69^ Copyright 2021, American Chemical Society.

Pt hollow nanodendrites with enhanced POD‐mimic ability were also reported for wound healing.^33^ Pt branches were synthesized on the Pd cores via a wet etching method (Figure 3A). The high‐index facets and high atomic efficiency endowed the Pt hollow nanodendrites with good POD‐mimicking ability. In vitro antibacterial experiment displayed that Pt hollow nanodendrites achieved 72% and 80% bacterial inactive rates for *S. aureus* and *E. coli*, respectively, by adding H_2_O_2_. When applied in a mouse model of *S. aureus*‐infected wounds, Pt hollow nanodendrites with addition of H_2_O_2_ effectively inhibited *S. aureus* growth and obtained nearly complete wound healing at the sixth‐day treatment (Figure 3B). Moreover, Ir nanoplates showed intrinsic POD‐like activity for antibacterial treatment by catalyzing the generation of •OH.^68^ Nanozymes with copper elements are highly valuable because of their excellent antimicrobial ability in biomedical field. In recent studies, copper‐doped nanozymes as POD mimic could catalyze H_2_O_2_ to produce •OH for treating wound infection (Figure 3C).^69^ As the copper‐doped nanozymes could inhibit 95% and 94% growth of *E. coli* and *S. aureus*, respectively, via releasing copper and generating ROS. The bacteria‐infected wounds were efficiently healed at the seventh day post‐treatment (Figure 3D). This study provided a strategy using copper‐element‐fabricated nanozymes with antimicrobial property for wound infection administration.

### 4.2 Binary nanozymes

**FIGURE 4:**
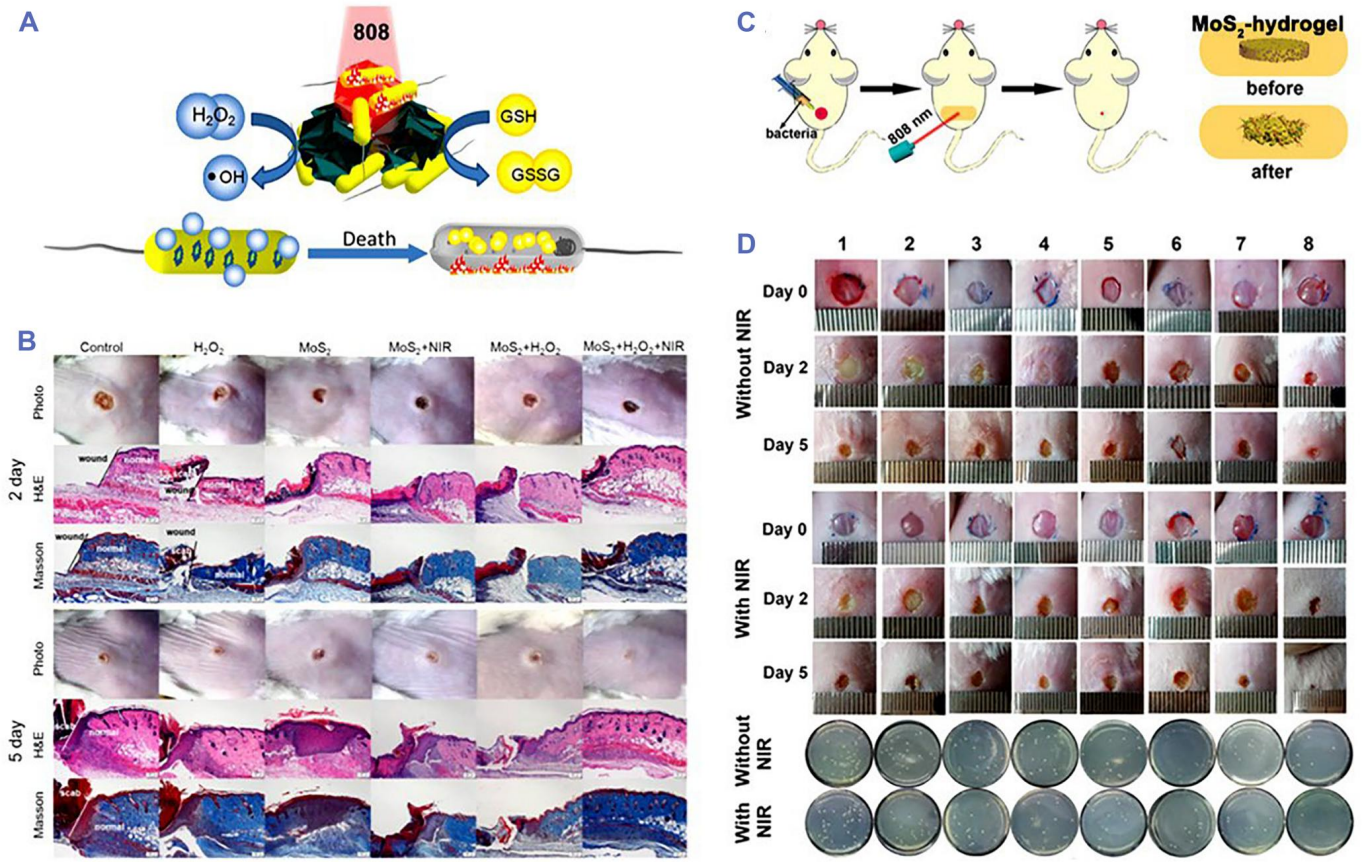
(A) Scheme of POD‐like MoS_2_ NFs with photothermal performance for killing bacteria. (B) Photographs of wounds and bacterial CFU in wound tissue treated by different groups. *Source*: Images reprinted with permission from Ref.^81^ Copyright 2016, American Chemical Society. (C) Scheme of MoS_2_‐hydrogel with POD‐like and photothermal performance for treating wound infection. (D) Photographs of wound and bacterial CFU in wound tissue treated by (1) phosphate buffer saline (PBS), (2) H_2_O_2_, (3) MoS_2_‐cysteine, (4) MoS_2_‐cysteine + H_2_O_2_, (5) hydrogel, (6) hydrogel + H_2_O_2_, (7) MoS_2_‐hydrogel, and (8) MoS_2_‐hydrogel + H_2_O_2_, respectively. *Source*: Images reprinted with permission from Ref.^84^ Copyright 2019, WILEY‐VCH Verlag GmbH & Co. KGaA, Weinheim. CFU, colony forming unit; NIR, near‐infrared

Metal chalcogenide compound nanomaterials are considerably important due to their special physicochemical, structural, and photothermal properties, endowing them the attractive applications in biomedicine.^70^, ^71^, ^72^, ^73^ Metal chalcogenide compound nanomaterials, such as Fe_3_O_4_ nanoparticles, CeO_2_ nanoparticles, Mn_3_O_4_ nanoparticles, and MoS_2_ nanomaterials, can mimic the activity of natural POD to convert H_2_O_2_ into •OH.^34^, ^72^, ^74^, ^75^, ^76^, ^77^, ^78^, ^79^, ^80^ Among them, MoS_2_ nanomaterials are probably the most common binary compounds and used to manage bacterial infections.^34^, ^81^, ^82^, ^83^ Yin et al. developed a POD‐like binary nanozyme using functionalized MoS_2_ nanoflowers (PEG‐MoS_2_ NFs) for synergetic catalytic and photothermal treatment of wound infection (Figure 4A).^81^ The PEG‐MoS_2_ NFs with photothermal and peroxidase‐mimicking properties could kill 54% *E. coli* and 87% *Bacillus subtilis* (*B. subtilis*) by adding H_2_O_2_, while their bacterial inhibition rate reached to 97% for *E. coli* and 100% for *B. subtilis* under NIR light irradiation for 10 min, which subsequently contributed to satisfying wound disinfection in mice. Overall, the synergistic antibacterial treatment by PEG‐MoS_2_ NFs could effectively manage bacterial infection and promote wound healing (Figure 4B).

In addition, Qu's group designed an MoS_2_‐based photomodulated nanozyme to realize Gram‐selective antimicrobial treatment.^34^ The MoS_2_‐based nanosystem with the peroxidase‐like activity inhibited the growth of 37% *E. coli* and 58% *S. aureus*, respectively, resulted from the combination of increasing positive charges and catalytic generation of •OH after exposure to 365 nm light. Meanwhile, the nanosystem achieved efficient treatment against two strains of bacteria‐induced wound infection, in which *S. aureus* infection required 10 min of irradiation and *E. coli* infection needed 25 min of irradiation. Based on the previous nanosystem, an MoS_2_ nanozyme‐hydrogel was developed to improve the ability of capturing and restricting bacteria (Figure 4C).^84^ Under laser irradiation, the MoS_2_‐hydrogel caused 95% and 97% inactivated rates for *S. aureus* and *E. coli*, respectively, in the presence of H_2_O_2_ and efficiently captured bacteria and realized wound disinfection in mice (Figure 4D). Recently, a defect‐rich MoS_2_ nanozyme was fabricated by the same group for effective bacteria killing and wound healing.^83^ Besides, Chu's group reported a nanozyme‐based stretchable hydrogel; an MoS_2_‐polydopamine nanozyme composite hydrogel (MPH) could eradicate bacterial infection after exposure to NIR laser and reduce oxidative stress for disinfection and wound healing.^85^

Apart from these developed MoS_2_ nanozymes, manganese dioxide (MnO_2_) nanozymes with the oxidase‐like activity showed fascinating advantages for treating various bacterial infections.^86^ The MnO_2_ nanozyme (8 mM) could inhibit 90% and 80% of *S. aureus* and *E. coli*, respectively, by catalyzing the production of ^1^O_2_ and •OH. Du et al. designed an iron oxide (Fe_3_O_4_)‐based nanozyme to regulate pathological microenvironment for promoted wound healing.^77^ The Fe_3_O_4_ nanozyme with POD‐like activity could covert H_2_O_2_ into •OH for bacterial death against *E. coli* and MRSA. Moreover, Fe_3_O_4_ nanozyme coating with glucose oxidase showed broad‐spectrum antimicrobial activity and efficiently promoted wound healing in diabetic ulcer. Gao et al. reported a bimetallic alloy nanocage (AgPd_0.38_) with oxidase‐like activity for wound healing.^87^ The AgPd_0.38_ nanozymes could selectively kill bacteria over mammalian cells by generating surface‐bound ROS with the minimum bactericidal concentration of 4–64 μg/ml to kill 99.9% bacterial cells (MBC_99.9_) against various bacteria. Moreover, the MBC_99.9_ of AgPd_0.38_ nanozymes was 16 μg/ml against the laboratory antibiotic‐sensitive *S. aureus* strain. In vivo animal experiments showed that the AgPd_0.38_ nanozymes could significantly promote wound healing against *P. aureginosa*‐infected wounds.

### 4.3 Ternary nanozymes

**FIGURE 5:**
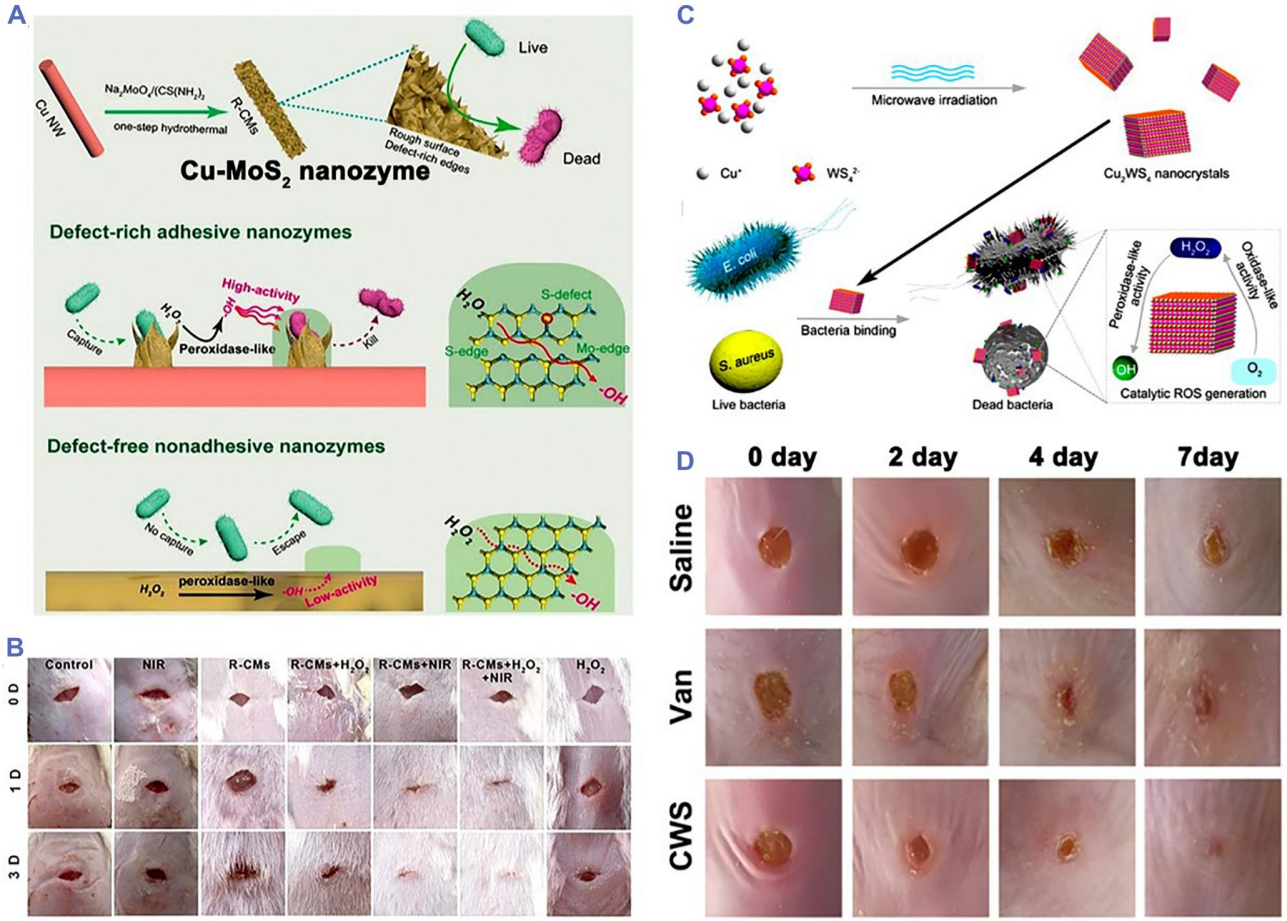
(A) Scheme of the synthesis process and antibacterial performance of Cu‐MoS_2_ nanozymes. (B) Photographs of wounds treated by different groups. *Source*: Images reprinted with permission from Ref.^83^ Copyright 2019, WILEY‐VCH Verlag GmbH & Co. KGaA, Weinheim. (C) Scheme of synthesis process and antibacterial performance of Cu_2_WS_4_ NPs (CWS) with dual enzyme‐like properties. (D) Photographs of wound treated by CWS and vancomycin (Van), respectively. *Source*: Images reprinted with permission from Ref.^35^ Copyright 2019, American Chemical Society. ROS, reactive oxygen species

Ternary metal chalcogenide compound nanomaterials played significant roles because of their unique physicochemical properties and adjustable compositions in biomedicine.^88^, ^89^ Ternary metal chalcogenides are composed of three elements, which have well‐defined crystal structures and can overcome the limitation of active sites.^88^, ^89^, ^90^, ^91^, ^92^ Thus, exploring the enzyme‐like and antibacterial properties of ternary metal chalcogenides is of particular significance. Recently, many ternary nanozymes were studied in biomedicine.^93^, ^94^, ^95^, ^96^, ^97^ For example, Cu‐MoS_2_ nanozymes were used as a defect‐rich antibacterial nanoagent for wound healing (Figure 5A).^83^ These Cu‐MoS_2_ nanozymes were demonstrated abundant rough surfaces to capture and kill bacteria by their defect‐rich edges and enhanced generation of ROS. Notably, the affinity ability of the defect‐rich MoS_2_ nanozymes for H_2_O_2_ was significantly higher than that of pristine MoS_2_. In mice with bacteria‐infected wounds, Cu‐MoS_2_ nanozymes with the POD‐mimic activity completely obliterated bacteria with the assistance of H_2_O_2_ and NIR laser (Figure 5B). Li et al. studied the Fe‐coated MoS_2_ nanosheets (MoS_2_/Fe) as dual nanozyme‐based ternary nanozymes for antibacterial treatment and wound healing.^98^ The MoS_2_/Fe nanozymes showed multifunctional properties, including glutathione loss, photothermal treatment, and •OH generation by POD‐mimic catalysis under acid condition. Benefitting from these properties, the MoS_2_/Fe nanozymes not only exhibited ∼100% bacterial inhibition rate with the assistance of H_2_O_2_ and NIR laser, but also presented an extraordinary advantage of bacterial inhibition, wound healing, and skin regeneration in vivo.

In particular, we first reported a ternary nanozyme with bacteria‐binding property to treat MRSA‐infected wounds (Figure 5C).^35^ The Cu_2_WS_4_ nanocrystals (CWS NCs) with dual enzyme‐mimic activities were synthesized by a microwave‐assisted method. CWS NCs could not only capture bacteria, but also showed oxidase‐ and peroxidase‐mimic properties to generate ROS including H_2_O_2_ and •OH without additional light irradiation, which resulted in >99.999% inactivation for *E. coli*, MRSA, and *S. aureus* at a concentration of 2 μg/ml. Notably, CWS NCs showed the higher antibacterial activity than most of the common antibacterial nanomaterials and antibiotics. Furthermore, CWS NCs effectively treated bacteria‐induced wound infection in mice (Figure 5D). Following on this study, we further developed an NIR‐II responsive ternary nanozyme (Cu_2_MoS_4_ nanoparticles, CMS NPs) to kill multidrug‐resistant bacteria.^99^ The CMS NPs displayed excellent NIR‐II photothermal conversion performance (37.8%), OXD‐like and POD‐like activity, and NIR‐II light improved the catalytic properties of dual nanozymes. When tested in vitro under NIR‐II light irradiation, 40 μg/ml of CMS NPs effectively caused 8 log inactivation for multidrug‐resistant *E. coli* and 6‐log inactivation for multidrug‐resistant *S. aureus*. Moreover, CMS NPs showed 6‐log inactivation for multidrug‐resistant *S. aureus* in vivo. Overall, these studies proved that Cu‐based ternary nanozymes have great potential to be applied in the field of wound infection.

## 5 MOF‐BASED NANOZYMES FOR WOUND HEALING

MOFs endowed with unique porous structure, adjustable pore size, high surface area, and permanent porosity are self‐assembled between inorganic units and organic linkers by strong coordination bonds, having attracted great attentions in biomedicine.^100^, ^101^, ^102^, ^103^, ^104^, ^105^, ^106^ In recent years, MOFs have been studied as the natural enzyme mimic and show similar catalytic mechanisms as natural enzymes for killing various strains of bacteria.^102^, ^103^, ^107^, ^108^ Zhang et al. developed a POD‐like nanozyme using Ag ion‐infused MOFs (AMOF) and the ability of converting H_2_O_2_ into •OH and releasing Ag ion for bacteria killing.^102^ Antibacterial experiments in vitro indicated that the survival rate of *E. coli* was 75% in AMOF‐treated group without the assistance of H_2_O_2_. The AMOF could completely kill bacteria with additional 10 mM H_2_O_2_. The different antibacterial effect of H_2_O_2_ could be correlated with different bacterial structures and isoelectric points.

**FIGURE 6:**
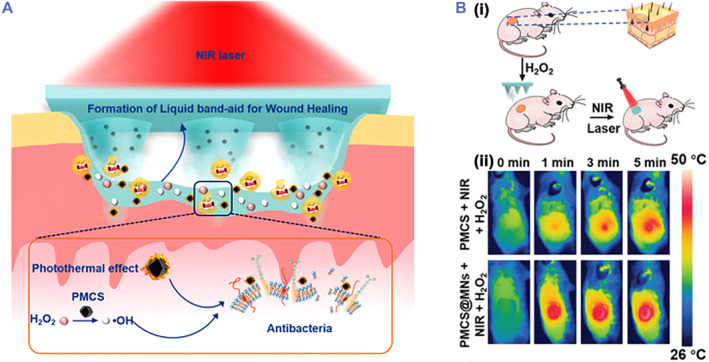
(A) Scheme of MOF‐derived multifunctional porphyrin‐like metal center NPs (PMCS) integrated microneedles (PMCS‐MNs) with the POD‐like activity for antibacterial treatment. (B) Scheme of PMCS‐MNs for wound treatment (i) and the near‐infrared (NIR) thermal images of wounds (ii). *Source*: Images reprinted with permission from Ref.^108^ Copyright 2021, Wiley‐VCH GmbH.

In other studies, Li et al. fabricated an MOF nanozyme composite cryogel (CSG‐M_X_) for antibacterial treatment.^103^ The CSG‐M_X_ not only showed excellent OXD‐ and POD‐like activities for catalyzing O_2_ and H_2_O_2_ to generate O_2_^•−^ and •OH, but also actively attacked bacteria because of their positive charges and inhibited bacteria with additional 100 μM H_2_O_2_. In vivo results further indicated that CSG‐M_1.5_ with 100 μM H_2_O_2_ could effectively reduce wound area and promote wound healing at pH ∼4. Sun et al. developed an NIR laser‐triggered MOF nanozyme to promote wound healing (Figure 6A).^108^ The MOF nanozymes loaded microneedle showed NIR photothermal conversion performance and POD‐like activity for catalyzing H_2_O_2_ to generate •OH. Benefiting from these properties, the microneedle showed good antibacterial performance in the presence of H_2_O_2_ and NIR laser and successfully downregulated the expression of proinflammatory factors and promoted wound healing in mice (Figure 6B).

## 6 HYBRID NANOZYMES FOR WOUND HEALING

**FIGURE 7:**
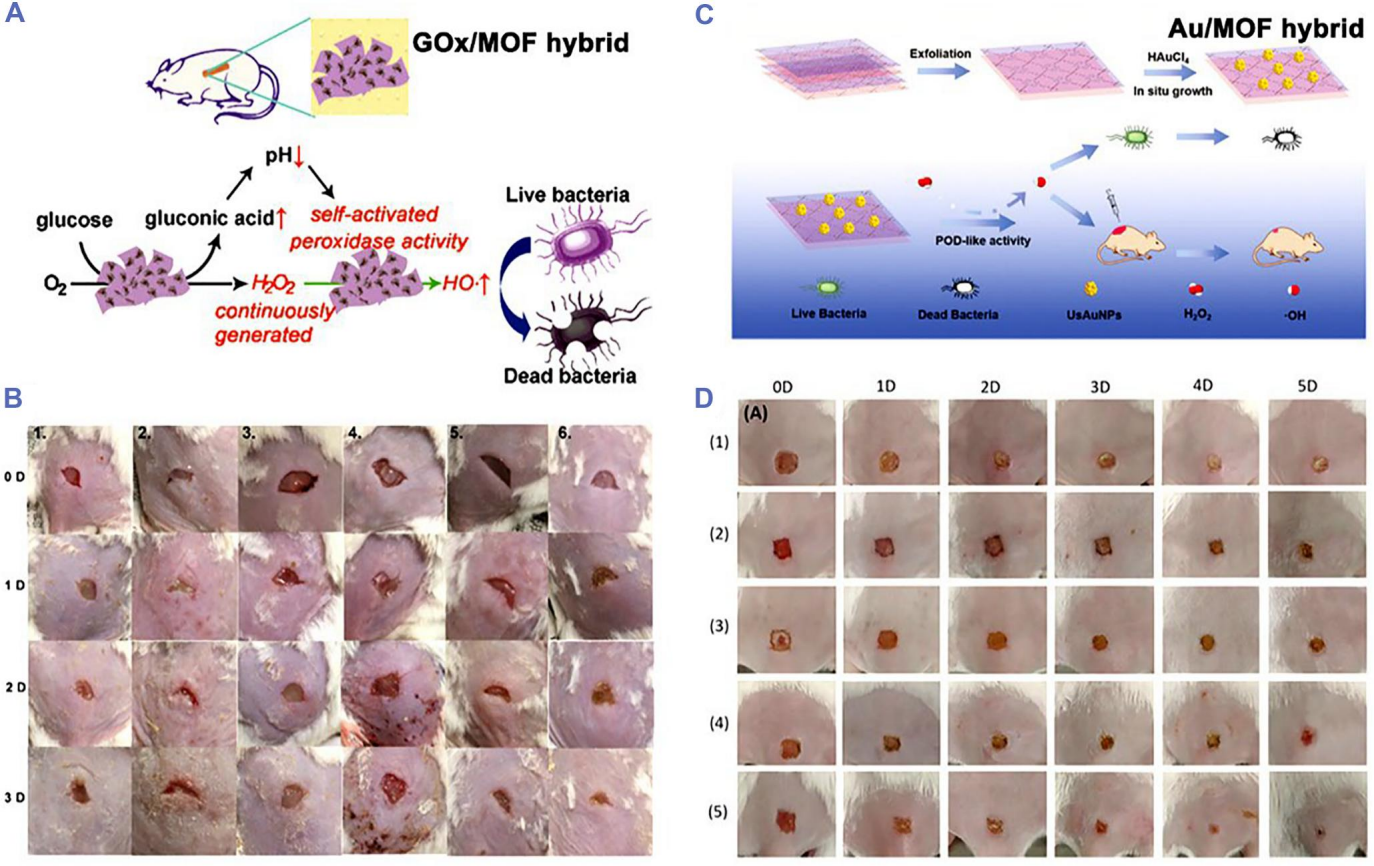
(A) Scheme of 2D MOF (Cu‐TCPP(Fe)) nanosheet absorbing glucose oxidase (GOx) as a hybrid nanozyme for antibacterial treatment. (B) Photographs of *S. aureus*‐infected wound treated by different groups. *Source*: Images reprinted with permission from Ref.^115^ Copyright 2019, American Chemical Society. (C) Scheme of the preparation process and antibacterial application of Au NPs/MOFs hybrid nanozyme. (D) Photographs of *S. aureus*‐infected wound treated by (1) phosphate buffer saline (PBS), (2) Au NPs/MOFs, (3) H_2_O_2_, (4) MOFs + H_2_O_2_, and (5) Au NPs/MOFs + H_2_O_2_, respectively. *Source*: Images reprinted with permission from Ref.^65^ Copyright 2020, WILEY‐VCH Verlag GmbH & Co. KGaA, Weinheim. MOF, metal organic framework

The development of nanozymes has been limited in clinical transformation due to their low catalytic activity. Hybrid nanozymes fabricated by different nanozymes or natural enzymes have demonstrated improved catalytic activities for biomedical applications.^109^, ^110^, ^111^, ^112^, ^113^, ^114^ Wang et al. reported a g‐C_3_N_4_@Au hybrid nanozyme (CNA) by using Au NPs to enhance the catalytic ability of g‐C_3_N_4_ nanosheets.^114^ CNA nanozymes showed higher POD‐mimicking catalytic performance than g‐C_3_N_4_ nanosheets and Au NPs, which could be attributed to their synergistic effect. With the assistance of 1 mM H_2_O_2_, the CNA nanozymes could almost completely kill drug‐resistant *E. coli* and MRSA by the catalytic production of •OH. In vivo animal experiments indicated that the CNA nanozymes efficiently killed bacteria and promoted wound healing. Liu et al. also provided a hybrid nanozyme integrating 2D MOF (POD mimic) with natural enzymes (glucose oxidase, GOx) for wound healing (Figure 7A).^115^ The MOF/GOx hybrid nanozyme first catalyzed glucose to continuously produce gluconic acid and H_2_O_2_, while the produced H_2_O_2_ was converted into •OH via the cascade reaction. In the presence of glucose, the bacterial inactivation rates of MOF/GOx hybrid nanozyme were 90% and 88% for *S. aureus* and *E. coli*, respectively, exhibited good antibacterial activity, and accelerated wound healing in vivo after the treatment (Figure 7B).

Recently, Hu et al. reported Au NPs/2D MOF hybrid nanozymes for antibacterial treatment (Figure 7C).^65^ The Au NPs/MOF nanozyme showed the POD‐like activity toward H_2_O_2_ conversion into •OH, contributing to effectively kill bacteria with additional H_2_O_2_ and wound healing in vivo (Figure 7D). Xi et al. designed a Cu/carbon hybrid nanozyme with the POD‐like activity that killed bacteria with a >1.2 log‐reduction in viability for *E. coli*, *S. aureus*, and *Streptococcus mutans* (*S. mutans)* by adding H_2_O_2_.^116^ Animal experiments further confirmed that Cu/carbon nanozyme efficiently treated bacteria‐infected animal models and promoted wound healing. Xiao et al. reported Fe_3_O_4_‐polydopamine (Fe_3_O_4_/PDA) hybrid nanozymes for wound disinfection.^37^ The Fe_3_O_4_/PDA nanozyme continuously catalyzed O_2_ to produce •OH via glutathione‐depleted cascade reactions, which not only could capture and kill bacteria, but also effectively treated bacteria‐infected wounds.

## 7 OTHER ANTIBACTERIAL NANOZYMES FOR WOUND HEALING

**FIGURE 8:**
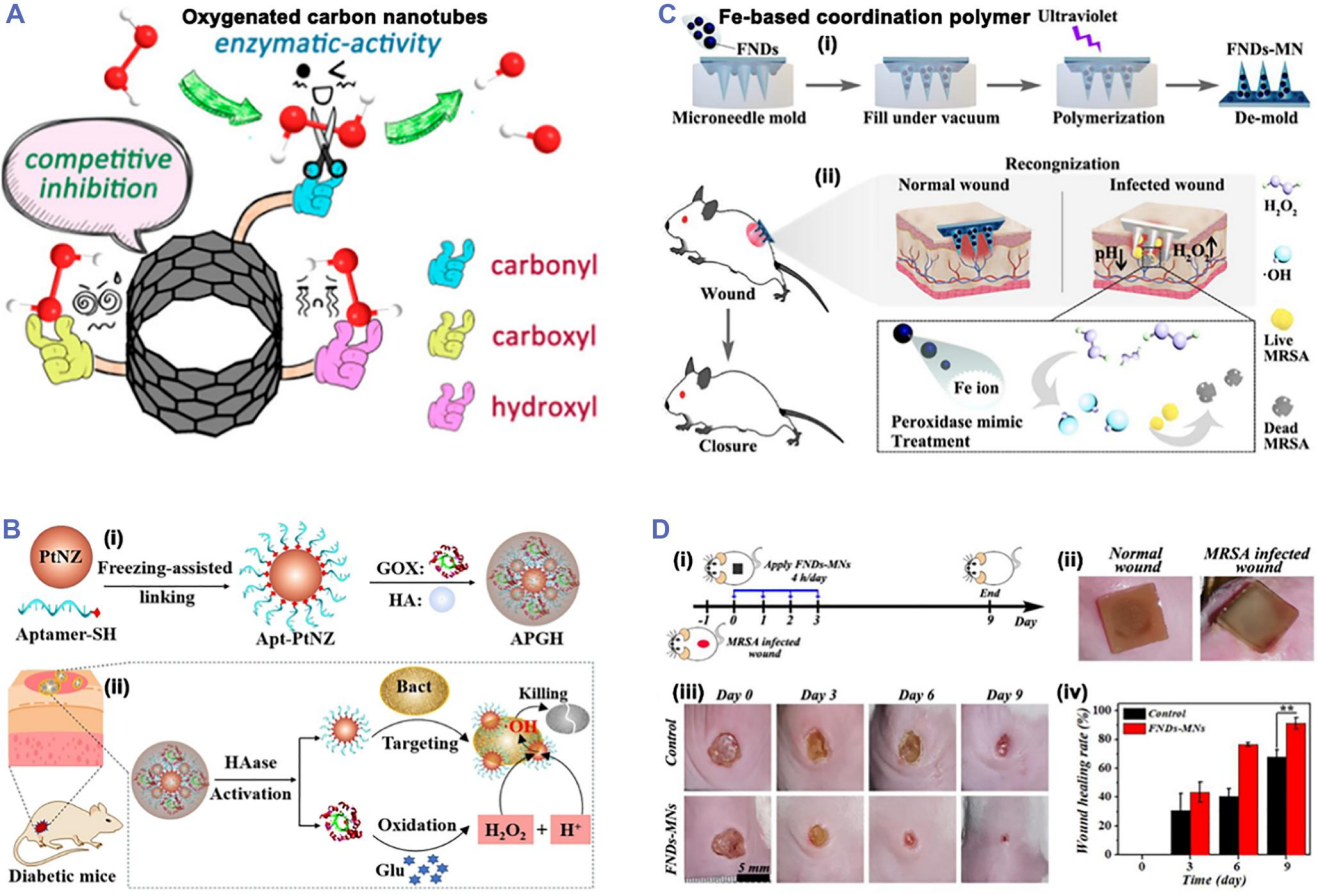
(A) Scheme of oxygenated carbon nanotubes with the enzyme‐like activity. *Source*: Images reprinted with permission from Ref.^122^ Copyright 2018, American Chemical Society. (B) Scheme of the synthesis process (i) and anti‐infection treatment (ii) for APGH. *Source*: Images reprinted with permission from Ref.^38^ Copyright 2021, Wiley‐VCH GmbH. (C) Scheme of FNDs‐MNs preparation process (i) and anti‐infection treatment (ii). (D) Scheme of the therapeutic process (i), MNs colorimetric photographs for different wounds (ii), FNDs‐MNs for treating MRSA‐infected wounds (iii), and wound healing conditions (iv). *Source*: Images reprinted with permission from Ref.^124^ Copyright 2022, Elsevier.

Apart from SAzymes, metal‐based, MOF‐based, and hybrid nanozymes, other antibacterial nanozymes have been also studied for wound care.^38^, ^64^, ^117^, ^118^, ^119^, ^120^ For instance, Xi et al. reported that sponge‐like carbon nanozyme with the POD‐like activity could kill bacteria and treat wound infection in vivo by the catalytic‐photothermal synergetic strategy.^121^ Wang et al. also reported oxygenated carbon nanotubes with the POD‐like activity for treating wound infections (Figure 8A).^122^ He et al. prepared bamboo‐like nitrogen‐doped carbon nanotubes encapsulating cobalt nanoparticles with the POD‐like activity for antibacterial treatment.^123^ In another study, aptamer‐functionalized Pt nanozymes and GOx were co‐caged in a hyaluronic acid shell (APGH) as a glucose activatable nanozyme for treating diabetic infections (Figure 8B).^38^ In the bacteria‐infected wounds, the HA shell of APGH could be degraded by hyaluronidase, followed by the release of Pt and GO_X_. The GOx further catalyzed glucose to generate gluconic acid and H_2_O_2_, which decreased microenvironment pH to accelerate the POD‐mimicking catalytic activity for enhanced generation of •OH. The APGH with 10 mM glucose could not only kill *S. aureus* within 4 h in vitro, but also effectively treat *S. aureus*‐infected wounds in diabetic mice. Moreover, our group recently developed an Fe‐based coordination polymer (FND) nanozyme to treat wound infection (Figure 8C).^124^ The FNDs with the POD‐like activity were prepared via the coordination reactions. The FNDs not only showed good antibacterial activity by catalyzing the generation of •OH in vitro, but also recognized different wounds with effective bacteria‐infected wound treatments after being loaded in microneedle patch for administration (Figure 8D).

## 8 CONCLUSIONS AND OUTLOOK

In the review, we summarize the advances of emerging antibacterial nanozymes against bacteria‐infected wounds. Antibacterial nanozymes can kill various bacteria and effectively treat wound infections by catalyzing the generation of ROS. Current reported antibacterial nanozymes mainly include noble metals, carbon‐based, transition metal‐based compounds, and MOF‐based nanomaterials (Table 1). However, most of them only show single enzyme‐mimic activity and limited antibacterial effect, which largely compromise their further clinical transformation. Thus, studies of antibacterial nanozymes, including mechanisms, antibacterial types, and biosafety, need to be further explored as the following aspects.

1. Catalytic mechanism of antibacterial nanozymes. Due to the difficulties in monitoring of the catalytic reaction in the antibacterial process, the catalytic mechanism of nanozymes remains poorly understood. An accurate comprehension of the catalytic mechanism will provide a guidance to regulate the catalytic activity and the therapeutic effect of antibacterial nanozymes and promote the design of new effective antibacterial nanozymes.
2. Types of antibacterial nanozymes. Natural enzymes can catalyze various biochemical reactions, but current antibacterial nanozymes only show limited redox and hydrolytic reactions (OXD or POD). Thus, it is significant to explore novel antibacterial nanozymes with the abilities to catalyze other types of biochemical reactions.
3. Biosafety assessment. Although nanozymes have showed a good antibacterial effect both in vivo and in vitro, the biosafety of nanozymes in the whole body needs to be further systematically studied before clinical transformation.
4. Smart antibacterial nanozymes. Current studies of antibacterial nanozymes mainly focus on the therapeutic effect. Other promising nanozymes with on‐demand treatment abilities need to be further explored, for example, microenvironment‐responsive regulation of ROS levels in the infected site to realize on‐demand treatments, nanozymes with colorimetric ability, and nanozymes‐based drug delivery.

**TABLE 1:** Summary of antibacterial nanozymes

Nanozymes	Enzyme mimic	Antibacterial mechanism	References
Zn‐based SAzyme	POD	Generation of •OH	53
Cu‐based SAzyme	POD	Photothermal + generation of •OH	32
Fe‐based SAzyme	POD	Generation of •OH	54
Mn‐based SAzyme	POD	Photothermal + generation of •OH	57
Pd‐based SAzyme	POD	NO + generation of •OH	56
Au NPs	OXD and POD	Generation of •OH	64
Pd nanocrystals	OXD and POD	Generation of H_2_O_2_ and •OH	67
Pt nanodendrites	POD	Generation of •OH	33
Cu nanozymes	POD	Generation of •OH	69
MoS_2_ nanoflowers	POD	Photothermal + generation of •OH	34, 81, 84
MnO_2_ nanozymes	OXD	•OH	86
Fe_3_O_4_ nanozymes	POD	Generation of •OH	77
AgPd_0.38_ nanozymes	OXD	Generation of ROS	87
Cu‐MoS_2_	POD	Photothermal + generation of •OH	83
MoS_2_/Fe NSs	POD	Photothermal + generation of •OH	98
Cu_2_WS_4_ NCs	OXD and POD	Generation of H_2_O_2_ and •OH	35
Cu_2_MoS_4_ NPs	OXD and POD	Generation of H_2_O_2_ and •OH	99
MOF	OXD and POD	Generation of O_2_^•−^ and •OH	103
g‐C_3_N_4_@Au hybrid	POD	Generation of •OH	114
MOF/GOx hybrid	OXD and POD	Generation of •OH	115
Au/MOF hybrid	POD	Generation of •OH	65
Cu/carbon hybrid	POD	Generation of •OH	116
Fe_3_O_4_/PDA hybrid	OXD and POD	Photothermal + generation of •OH	37
Polymer	POD	Generation of •OH	124

## AUTHOR CONTRIBUTIONS

Jingyang Shan and Yuanjin Zhao conceived the idea; Jingyang Shan wrote the paper; Junyi Che and Chuanhui Song revised the paper.

## CONFLICT OF INTEREST STATEMENT

The authors declare no conflict of interest.
